# Predictors for hemostatic thrombin-gelatin matrix usage in spine surgery: a multicenter observational study

**DOI:** 10.1186/s12891-023-06408-8

**Published:** 2023-04-13

**Authors:** So Kato, Junya Miyahara, Yoshitaka Matsubayashi, Yuki Taniguchi, Toru Doi, Hiroyasu Kodama, Akiro Higashikawa, Yujiro Takeshita, Masayoshi Fukushima, Takashi Ono, Nobuhiro Hara, Seiichi Azuma, Hiroki Iwai, Masahito Oshina, Shurei Sugita, Shima Hirai, Kazuhiro Masuda, Sakae Tanaka, Yasushi Oshima, University of Tokyo Spine Group

**Affiliations:** 1 Department of Orthopaedic Surgery, the University of Tokyo, 7-3-1 Hongo, Bunkyo-ku, Tokyo, 113-8655 Japan; 2 Department of Spine and Orthopedic Surgery, Japanese Red Cross Medical Center, 4-1-22 Hiroo, Shibuya-ku, Tokyo, 150-8935 Japan; 3 Department of Orthopedic Surgery, Japan Organization of Occupational Health and Safety Kanto Rosai Hospital, Kizukisumiyoshi- cho, Nakahara-ku, Kawasaki, 211-8510 Japan; 4 Department of Orthopedic Surgery, Organization of Occupational Health and Safety Yokohama Rosai Hospital, 3211 Kozukue-Chō, Kōhoku-Ku, 222-0036 Yokohama, Japan; 5 Spine center, Toranomon Hospital, 2-2-2 Toranomon, Minato-ku, 105-8470 Tokyo, Japan; 6 Department of Spinal Surgery, Community Health-Care Organization Tokyo Shinjuku Medical Center, 5-1 Tsukudo-cho, Shinjuku-ku, 162-8543 Tokyo, Japan; 7 Department of Orthopedic Surgery, Japanese Red Cross Musashino Hospital, 1-26-1 Kyonancho, Musashino, 180-8610 Tokyo, Japan; 8 Department of Orthopedic Surgery, Saitama Red Cross Hospital, 1-5 Shintoshin, Chuo-ku, 330-8553 Saitama, Japan; 9 Iwai Orthopaedic Medical Hospital, 8-17-2 Minamikoiwa Edogawa-ku, 133- 0056 Tokyo, Japan; 10 Department of Orthopedic Surgery, NTT Medical Center Tokyo, 5-9-22 Higashi- Gotanda, Shinagawa-ku, 141-8625 Tokyo, Japan; 11 Department of Orthopedic Surgery, Tokyo Metropolitan Cancer and Infectious Diseases Center Komagome Hospital, 3-18-22 Honkomagome, Bunkyo-ku, 113-8677 Tokyo, Japan; 12 Department of Orthopedic Surgery, Sagamihara National Hospital, 18-1 Sakuradai, Minami-ku, 252-0392 Sagamihara, Kanagawa Japan; 13 Department of Orthopedic Surgery, Tokyo Metropolitan Tama Medical Center, 2-8-29 Musashidai, Fuchu, 183-8524 Tokyo, Japan

**Keywords:** Thrombin-gelatin matrix, Multicenter study, Risk factor, Predictor, Logistic regression analysis

## Abstract

**Study design:**

A prospective cohort study.

**Objectives:**

Thrombin-gelatin matrix (TGM) is a rapid and potent hemostatic agent, but it has some limitations, including the cost and its preparation time. The purpose of this study was to investigate the current trend in the use of TGM and to identify the predictors for TGM usage in order to ensure its proper use and optimized resource allocation.

**Methods:**

A total of 5520 patients who underwent spine surgery in a multicenter study group within a year were included in the study. The demographic factors and the surgical factors including spinal levels operated, emergency surgery, reoperation, approach, durotomy, instrumented fixation, interbody fusion, osteotomy, and microendoscopy-assistance were investigated. TGM usage and whether it was routine or unplanned use for uncontrolled bleeding were also checked. A multivariate logistic regression analysis was used to identify predictors for unplanned use of TGM.

**Results:**

Intraoperative TGM was used in 1934 cases (35.0%), among which 714 were unplanned (12.9%). Predictors of unplanned TGM use were female gender (adjusted odds ratio [OR]: 1.21, 95% confidence interval [CI]: 1.02–1.43, p = 0.03), ASA grade ≥ 2 (OR: 1.34, 95% CI: 1.04–1.72, p = 0.02), cervical spine (OR: 1.55, 95% CI: 1.24–1.94, p < 0.001), tumor (OR: 2.02, 95% CI: 1.34–3.03, p < 0.001), posterior approach (OR: 1.66, 95% CI: 1.26–2.18, p < 0.001), durotomy (OR: 1.65, 95% CI: 1.24–2.20, p < 0.001), instrumentation (OR: 1.30, 1.03–1.63, p = 0.02), osteotomy (OR: 5.00, 2.76–9.05, p < 0.001), and microendoscopy (OR: 2.24, 1.84–2.73, p < 0.001).

**Conclusions:**

Many of the predictors for unplanned TGM use have been previously reported as risk factors for intraoperative massive hemorrhaging and blood transfusion. However, other newly revealed factors can be predictors of bleeding that is technically challenging to control. While routine usage of TGM in these cases will require further justification, these novel findings are valuable for implementing preoperative precautions and optimizing resource allocation.

## Introduction

As the surgical techniques of spinal surgery have drastically evolved over the past decades, with an increasing number of invasive procedures indicated for more vulnerable patients than ever, the concept of patient blood management to optimize the patient outcome has been recognized in spinal surgery. Among the three pillars of patient blood management, which are the optimization of the red blood cell mass, blood loss, and anemia, [1] the improved control of intraoperative bleeding has been sought through meticulous hemostatic procedures and the use of modern hemostatic agents.

Intraoperative hemostatic agents can be classified into passive *versus* active agents. Passive agents such as bone wax, surgical cellulose, collagen, essentially function as mechanical blockade and/or provide the surface structure on which the coagulation cascade runs. On the contrary, thrombin-gelatin matrix (TGM) is an active hemostatic agent that has particularly rapid and potent efficacy in controlling massive bleeding. TGM is typically provided as the mixture of gelatin granules and thrombin solutions with a flowing property that is useful for various surfaces. With its local tamponade effect in conjunction with activation of coagulation cascade even in patients who are anticoagulated, have coagulation abnormalities and on antiplatelets, it has shown clinical benefits in many surgical specialty fields [2], including spinal surgery [3–8]. However, TGM has several limitations, including the cost and its preparation time. First, the typical dosage of TGM used in spinal surgery costs approximately $398 US per 10 cc unit, [9] with its price depending on jurisdiction; this makes it over 10 times more expensive than conventional passive hemostatic agents [4]. As such, its routine usage is not advisable from a health-economic perspective, particularly in settings where medical resources are limited. Second, TGM tends to be prepared on demand for intra-operative bleeding, as opposed to routine preparation given its cost. After being unpacked, thrombin is dissolved in saline and mixed with the gelatin matrix in a canister. The preparation time required may depend on the users’ experience, but the whole process could take up to 3 min, [10, 11] and in order to respond to massive intraoperative bleeding in a timely manner, TGM must be stored on-site in the operating room instead of in storage.

Therefore, it is of absolute importance to capture the current trend and establish justifiable indications of TGM usage in spinal surgery in order to optimize the patient outcomes as well as cost-effectiveness. The present study thus investigated the use of TGM by revealing real-world data from a multicenter study group and identified predictors of TGM usage to ensure its proper use and optimized resource allocation.

## Methods

### Patient samples and data collection

A prospective multicenter spine surgery registry has been carried out among one academic institution and 13 affiliated medical institutions located in an urban area and the database from April 2020 to March 2021 were utilized in the study. The diagnoses and surgical decision-making were made by board-certified spine surgeons based on physical examinations as well as imaging studies such as CT and MRI. The following demographic factors were collected: age, gender, body mass index, American Society of Anesthesiologists (ASA) physical status classification, diabetes, smoking, rheumatoid arthritis, and preoperative anticoagulation. The patients’ diagnosis were categorized into six groups: “degenerative disease”, “ligamentous ossification”, “deformity”, “tumor”, “trauma”, and “infection”. Regarding surgical procedures, the spinal levels operated, surgical approach, and whether the surgery was on an emergency basis or reoperation were investigated. Technical details obtained included durotomy, instrumented fixation, interbody fusion, osteotomy, and microendoscopy-assisted surgery (microendoscopic laminotomy, discectomy and transforaminal endoscopic lumbar interbody fusion) [12]. Surgical time and estimated intra-operative blood loss were also reported.

In the present study, the focus was given to TGM usage. Intra-operative TGM usage was determined at the operating surgeons’ discretion. In addition to the inquiry regarding the intra-operative usage of TGM, its indication was also investigated. The positive response to TGM usage inquiry did not preclude the concomitant usage of other passive hemostatic agents. The respondents were asked to choose whether it was unexpectedly used on an emergency basis reactively for uncontrollable bleeding where TGM was not preemptively prepared versus it was routinely used for obtaining perfect control of intra-operative bleeding. This reactive approach was documented as “unplanned” usage of TGM for further analyses.

### Statistical analyses

Baseline characteristics were compared between the groups using Student’s *t*-test or Mann-Whitney U test for continuous variables and the chi-square test or Fisher’s exact test for categorical variables. A multivariate logistic regression analysis was performed to identify its predictors for TGM usage. First, a univariate analysis was performed to identify candidate variables as predictors of TGM usage. Second, the variables considered relevant in the first stage as well as known risk factors for intraoperative massive bleeding and peri-operative transfusion previously reported in the literature were entered into a multivariable logistic regression model. Adjusted odds ratios with 95% confidence intervals were calculated. Correlations between the variables were tested by either Pearson’s correlation coefficient or Spearman’s rank correlation coefficient rho.

The comparisons between the unplanned TGM usage group and the routine TGM usage group were made to capture the demographic differences. The multivariate analysis to determine the predictors for unplanned use of TGM was further performed using the predictive factors for TGM usage as well as demographic factors that stood out to characterize the unplanned usage group.

All analyses were carried out using the IBM SPSS Statistics software program, version 26 (IBM Corp., Armonk, NY, USA). For all statistical tests, values of p < 0.05 were considered significant.

### Ethics approval and consent to participate

Informed consent was obtained from all participants, and approval for this study was given by the institutional review board of the Clinical Research Support Center at the University of Tokyo.

## Results

A total of 5520 patients who underwent spine surgery consecutively enrolled in the registry were included for the analysis. The mean age was 63.5 years old (range: 2–97, standard deviation: 17.9), and males accounted for 59.9% of the patients. A total of 82.1% of patients had an ASA physical status classification of ≥ 2. Other demographic data are summarized in Table 1. Intraoperative TGM was used in 1934 of the 5520 total cases included (35.0%). No specific complications directly related to TGM usage was reported in the present cohort. Comparisons of demographic factors between cases in which TGM was and was not used revealed that older patients, female patients, patients with a higher ASA grade, and patients with rheumatoid arthritis were more likely to receive intraoperative TGM than others (Table 1).

**Table 1:** Demographic data and univariate analyses

	Total	TGM used	No TGM used	p
n	5520	1934	3586	
Age (yrs)	63.5 (17.9)	65.2 (16.2)	63.0 (17.4)	< 0.001
Sex (male, %)	59.9%	57.8%	60.9%	0.03
BMI	23.9 (4.2)	24.0 (4.2)	24.0 (4.0)	0.11
Smoking	10.6%	10.4%	10.7%	0.69
ASA				
1	17.9%	14.5%	19.6%	
2	69.1%	73.6%	66.7%	
3	12.8%	11.6%	13.5%	
4	0.1%	0.1%	0.1%	
ASA ≥ 2	82.1%	85.4%	80.4%	< 0.001
Diabetes mellitus	18.2%	17.8%	18.5%	0.57
Rheumatoid arthritis	2.3%	3.5%	1.6%	< 0.001
Anticoagulation	15.8%	15.3%	16.0%	0.45

TGM: thrombin-gelatin matrix, BMI: body mass index, ASA: American Society of Anesthesiologists physical status classification

Continuous variables are shown as means and standard deviations in parentheses

The mean operation time was 147 (standard deviation [SD]: 103) minutes, and the median blood loss was 50 (interquartile range: 145) mL. The mean number of levels operated was 3.2 (SD: 2.0) levels. Comparisons between the two groups showed that number of levels operated, cervical spine, degenerative disease, ligamentous ossification, tumor surgery, posterior approach, durotomy, instrumentation, interbody fusion, and osteotomy were associated with an increased likelihood of TGM usage (Table 2). Usage rate of TGM ranged from 31.0% in the patients who underwent microendoscopic surgery to 71.2% in those undergoing osteotomy procedures.

**Table 2:** Comparisons of surgical factors according to TGM usage

	Total	TGM used	No TGM used	p
n	5520	1934	3586	
*Spinal level*				
Cervical	19.2%	21.8%	17.8%	< 0.001
Thoracic	13.7%	14.2%	13.5%	0.49
Lumbar	74.9%	71.9%	76.5%	< 0.001
*Diagnosis*				
Degenerative	73.2%	74.9%	72.3%	0.04
Trauma	7.1%	5.1%	8.2%	< 0.001
Infection	3.8%	1.9%	4.8%	< 0.001
Tumor	3.3%	4.4%	2.8%	0.001
Ligamentous ossification	2.6%	3.6%	2.0%	< 0.001
*Surgical factors*				
Anterior approach	4.3%	4.9%	4.0%	0.14
Posterior approach	84.5%	89.8%	81.6%	< 0.001
Number of levels operated	3.2 (2.0)	3.4 (2.0)	3.1 (2.0)	< 0.001
Emergency	11.1%	10.3%	11.5%	0.17
Revision	15.7%	15.5%	15.8%	0.71
Durotomy	6.8%	9.5%	5.4%	< 0.001
Fusion	35.4%	40.2%	32.8%	< 0.001
Interbody fusion	16.7%	21.7%	14.1%	< 0.001
Osteotomy	1.1%	2.2%	0.5%	< 0.001
Microendoscopy	35.2%	31.1%	37.4%	< 0.001

TGM: thrombin-gelatin matrix

Continuous values are shown in mean with standard deviation in parentheses

Based on the results of the univariate analyses as well as previous reports in the literature concerning predictors of intraoperative bleeding or transfusion, [13–17] the age, sex, ASA score, presence of rheumatoid arthritis, number of levels operated, and presence of cervical spine surgery, surgery for ligamentous ossification, tumor, infection, trauma, posterior approach, durotomy, instrumentation, osteotomy, and microendoscopy were selected as candidate predictors in the multivariate analysis, after deleting several factors based on the multicollinearity analysis results. The results of the multivariate analysis are summarized in Table 3. An ASA grade ≥ 2, rheumatoid arthritis, cervical spine, tumor, ligamentous ossification, posterior approach, durotomy, instrumentation, and osteotomy were found to be predictors of TGM usage, while trauma and microendoscopy were associated with a lower likelihood of TGM usage.

**Table 3:** Multivariate analysis for predictors of thrombin-gelatin matrix usage

Factor	Details	Adjusted odds ratio	95% CI		p
			Lower	Upper	
Age	Per 10y	1.03	0.99	1.07	0.17
Female		1.10	0.97	1.24	0.13
ASA ≥ 2		1.20	1.00	1.43	0.045
Rheumatoid arthritis		2.40	1.61	3.58	< 0.001
Number of levels operated	Per 1 level	0.99	0.95	1.02	0.40
Cervical spine		1.26	1.07	1.47	0.005
Trauma		0.72	0.55	0.95	0.02
Deformity		1.18	0.88	1.59	0.27
Infection		0.66	0.40	1.07	0.09
Tumor		1.45	1.04	2.02	0.03
Ligamentous ossification		1.43	1.01	2.04	0.04
Posterior approach		1.69	1.36	2.09	< 0.001
Durotomy		1.42	1.14	1.79	0.002
Instrumentation		1.18	1.02	1.36	0.03
Osteotomy		3.29	1.79	6.08	< 0.001
Microendoscopy		0.78	0.67	0.90	< 0.001

ASA: American Society of Anesthesiologists Classification, CI: confidence interval

TGM was unexpectedly used for uncontrollable bleeding in 714 cases, showing that TGM usage in the present cohort was routinely planned in 63.1% of cases. The comparisons of demographic data between the unplanned TGM usage group and the routine usage group is shown in Table 4. In summary, the unplanned TGM usage groups contained more anticoagulated patients (18.1% vs. 13.6%, p = 0.008), spinal deformity patients (7.8% vs. 5.2%, p = 0.02), osteotomy (3.6% vs. 1.3%, p < 0.001), microendoscopy (44.1% vs. 23.5%, p < 0.001), but less interbody fusion (19.0% vs. 23.2%, p = 0.03) than the routine TGM usage group. Based on the multivariate analysis results, age, sex and 11 predictors of TGM usage as well as anticoagulation, deformity and interbody fusion were selected as candidate variables for the multivariate analysis to determine the predictors of unplanned TGM. The identified predictors were female gender (adjusted odds ratio [OR]: 1.21, 95% confidence interval [CI]: 1.02–1.43, p = 0.03), ASA grade ≥ 2 (OR: 1.34, 95% CI: 1.04–1.72, p = 0.02), cervical spine (OR: 1.55, 95% CI: 1.24–1.94, p < 0.001), tumor (OR: 2.02, 95% CI: 1.34–3.03, p < 0.001), posterior approach (OR: 1.66, 95% CI: 1.26–2.18, p < 0.001), durotomy (OR: 1.65, 95% CI: 1.24–2.20, p < 0.001), instrumentation (OR: 1.30, 1.03–1.63, p = 0.02), osteotomy (OR: 5.00, 2.76–9.05, p < 0.001), and microendoscopy (OR: 2.24, 1.84–2.73, p < 0.001) (Table 5).

**Table 4:** Comparisons between the unplanned thrombin-gelatin matrix usage group and the routine usage group

	Unplanned TGM usage	Routine TGM usage	p
n	714	1220	
Age (yrs)	64.9 (16.5)	65.4 (16.1)	0.76
Sex (male, %)	55.3%	59.3%	0.08
BMI	24.3 (4.3)	23.9 (4.2)	0.11
Smoking	11.9%	9.5%	0.10
ASA ≥ 2	85.4%	85.5%	0.97
Diabetes mellitus	17.5%	18.0%	0.77
Rheumatoid arthritis	2.8%	3.9%	0.22
Anticoagulation	18.1%	13.6%	0.008
Number of levels operated	3.5 (2.3)	3.3 (1.9)	0.83
Cervical	20.0%	22.8%	0.16
Thoracic	15.8%	13.2%	0.11
Lumbar	72.7%	71.4%	0.54
Degenerative	73.0%	76.0%	0.14
Trauma	5.0%	5.1%	0.97
Deformity	7.8%	5.2%	0.02
Infection	1.4%	2.2%	0.21
Tumor	5.3%	3.9%	0.13
Ligamentous ossification	2.9%	4.0%	0.22
Anterior approach	4.1%	5.3%	0.21
Posterior approach	89.4%	90.1%	0.61
Emergency	9.5%	10.7%	0.40
Revision	17.1%	14.5%	0.13
Durotomy	10.6%	8.8%	0.17
Instrumentation	37.4%	41.9%	0.05
Interbody fusion	19.0%	23.2%	0.03
Osteotomy	3.6%	1.3%	< 0.001
Microendoscopy	44.1%	23.5%	< 0.001

TGM: thrombin-gelatin matrix, BMI: body mass index, ASA: American Society of Anesthesiologists Classification

**Table 5:** Multivariate analysis for predictors of unplanned thrombin-gelatin matrix usage

Factor	Details	Adjusted odds ratio	95% CI		p
			Lower	Upper	
Age	Per 10y	1.04	0.98	1.09	0.21
Female		1.21	1.02	1.43	0.03
ASA ≥ 2		1.34	1.04	1.72	0.02
Rheumatoid arthritis		1.28	0.78	2.10	0.33
Anticoagulation		1.11	0.95	1.31	0.20
Cervical spine		1.55	1.24	1.94	< 0.001
Trauma		0.85	0.58	1.24	0.39
Deformity		1.39	0.96	1.99	0.08
Tumor		2.02	1.34	3.03	< 0.001
Ligamentous ossification		1.30	0.80	2.13	0.29
Posterior approach		1.66	1.26	2.18	< 0.001
Durotomy		1.65	1.24	2.20	< 0.001
Instrumentation		1.30	1.03	1.63	0.02
Interbody fusion		1.09	0.82	1.44	0.57
Osteotomy		5.00	2.76	9.05	< 0.001
Microendoscopy		2.24	1.84	2.73	< 0.001

ASA: American Society of Anesthesiologists Classification, CI: confidence interval

## Discussion

Since TGM usage as an option for intraoperative hemostasis is associated with a higher cost than with conventional passive hemostatic agents, when utilizing thrombin, which is a blood product with limited availability, the optimized allocation is of absolute importance from a sociomedical perspective. This entails understanding the cases in which TGM is likely to be used based on previous trends as well as the determination of appropriate indications for TGM usage in spine surgery. To our knowledge, the present study is the first to shed light on the reality of intraoperative TGM usage in spine surgery and further elucidated its predictors in order to facilitate its efficient preparation. The results revealed that TGM was used in 35.0% of cases in a multicenter study cohort, and that female gender, cervical spine, tumor, posterior approach, durotomy, instrumentation, osteotomy, and microendoscopy were predictors of its unplanned usage.

The history of TGM is relatively short and a knowledge gap remains with regard to the real-world data concerning its usage in spinal surgery [18]. In 2015, Price et al. reported that they identified 40,057 cases with TGM usage (5.0%) among 807,280 cases registered in the United States Premier Perspective Hospital Database between 2006 and 2013, which was the inaugural phase for TGM commercialization [19]. However, more recent studies have reported higher percentage of TGM use. For example, Chen, et al. described 197 cases of thoracolumbar surgery with TGM among 293 total cases (67.2%) [6]. The previously reported rate of TGM usage has varied also depending on the surgical procedure applied, with the range being 5− 84% [3, 6–8, 19]. The most reasonable explanation for this discrepancy is the fact that the indication for TGM usage also varies. TGM is especially effective in severe to life threatening bleeding with amount of more than 10.0 mL/minute, described as “controllable spurting and/or overwhelming flow” to “unidentified or inaccessible spurting or gush”, which is categorized as Grade 3 or 4 in Validated Intraoperative Bleeding Scale (VIBe SCALE) [20]. However, it also plays an important role in obtaining complete control of Grade 1 to 2 bleeding, described as “ooze or intermittent flow” to “continuous flow”, for which meticulous hemostasis is required in spine surgery to prevent post-operative complications including epidural hematoma. Therefore, the TGM usage seems to be popularized in two polarized cohorts: moderately to highly invasive procedures expecting massive blood loss, and minimally invasive procedures with the risk of small but difficult to control bleeding. When it comes to the global trend, the TGM availability and economic burden associated in individual society may also play an important role to represent the frequency of usage. The cohort described in the present study represents the basic reality in a high-volume center of an urban area in a developed country while intraoperative decision-making concerning TGM usage was ultimately left to surgeons’ discretion.

The predictors for TGM usage identified in the present study were ASA grade ≥ 2, rheumatoid arthritis, cervical spine, tumor, ligamentous ossification, posterior approach, durotomy, instrumentation, and osteotomy. Many of these factors have been previously recognized as risk factors for massive intraoperative bleeding or perioperative transfusion usage. For example, Nuttall et al. reported that the determinants of an increased volume of allogenic blood transfused after spine surgery included tumor surgery and an increased number of posteriorly fused levels [16]. Regarding technical variations, Cha et al. reported that instrumented fusion was associated with the usage of allogenic blood transfusion in addition to autologous blood, [15] and Yu et al.’s study further revealed that osteotomy was a predictor of intraoperative massive blood loss in instrumentation surgery for scoliosis [14]. Furthermore, several studies have indicated that ossification of posterior longitudinal ligament (OPLL) was associated with a higher risk of intraoperative massive blood loss than cervical spondylotic myelopathy [17]. The mechanism underlying the bleeding tendency in OPLL has not been elucidated, but some authors have argued that altered circulation in the epidural venous plexus by ossification may be a possible explanation [21]. These factors would have not only directly led to increased intraoperative blood loss but also provoked an increased awareness of anticipated intraoperative bleeding among surgeons, leading to routine usage of TGM for managing the risk of continuous hidden blood loss and subsequent epidural hematoma formation. Indeed, more than 60% of cases of TGM usage fell in this category within the present study cohort.

It is worth noting, however, that some of the predictors for unplanned usage of TGM, such as cervical spine surgery, durotomy, and microendoscopy-assisted surgery, have not been previously acknowledged as risk factors neither for massive blood loss or transfusion. These newly revealed factors may represent risk factors for unexpected intraoperative bleeding that is technically challenging to control. For example, the cervical spine has several anatomical features that may be disadvantageous for achieving hemostasis. In the posterior cervical approach, the dural sac containing the spinal cord and cervical nerve roots are firmly tethered and cannot be effectively retracted for cauterization of the epidural venous plexus or application of solid hemostatic agents, in contrast to a posterior approach to the lumbar spine. The anterior cervical approach also has unique anatomical challenges. For example, vertebral arteries and surrounding veins can be sources of intractable bleeding, and continuous oozing from the vertebral foramens is frequently encountered, making conventional hemostatic techniques the suboptimal solution. The advantages of TGM in cervical spine procedures have previously been advocated, and the usage of these potent hemostatic agent can be justified, regardless of the actual volume of blood loss [3, 22]. Several previous studies have indicated that incidental durotomy was associated with increased blood loss [23–26]. This may be due in part to the prolonged operation time needed for dural repair, but other authors have reported that the loss of expansive tension of the dural sac due to cerebrospinal fluid leakage leads to the dilatation of the epidural venous plexus, [27] which can result in increased blood loss, necessitating unplanned TGM usage. Microendoscopy-assisted surgery is another procedure associated with technical difficulty, where surgeons have limited access to the source of bleeding. The usefulness of TGM in minimally invasive surgery has been reported, and two studies have described its usage as being associated with a significant reduction of risk of postoperative epidural hematoma, [7, 8] while a more recent randomized controlled trial found no marked benefit in preventing postoperative epidural hematoma, thereby discouraging the routine usage of TGM following microendoscopic surgery [28].

Several limitations associated with the present study warrant mention. First, the indication of TGM preparation and usage was solely left to surgeons. Therefore, the present results should only be considered to represent the current trends in urban practice and may not have accurately captured the patient and procedure characteristics of unpredictable intraoperative bleeding. In addition, even though the present results were obtained from multiple institutions, they may not be applicable to clinical practice in populations with different backgrounds. In the present study, the specific value of blood loss as a cut-off for the usage of TGM was not identified. Our study was designed to retrospectively capture the real-world data and TGM usage was expected to result in decreased blood loss. In addition, the information of TGM dosage used in surgery was not available. For example, 5 mL kit is commercially available for Floseal® (Baxter Healthcare Corp, Deerfield, IL) and it has been generally thought appropriate for standard spine surgery, but it does not prohibit surgeons from using larger dose as needed based on the extension of surgery or the severity of hemorrhage. Second, the present study did not include cost-effective analysis. Ianitti et al. reported lower bleeding-related complication rates with use of active hemostatic agents in comparison to combined active and passive agents while hospital costs decreased in United States and suggests proactive rather than counter-active usage of TGM during high grade bleeding in VIBe scale [29]. Wu et al. reported that despite the increased acquisition cost of TGM, the overall hospital cost for lumbar surgery was unchanged in comparison to cellulose and/or collagen [30]. Further investigations are warranted to elucidate in which specific cases TGM usage was justifiable and cost-effective among TGM group that accounted 35% of the present cohort since the direct cost of purchasing product is high and the present investigation was not designed as a health economic and outcomes research. Lastly, the impact of preparation time required for TGM was not investigated. With the development of newer mixture kit available for commercial TGM agents, the preparation time can be minimized and this could ultimately affect surgeons’ decision-making.

Nonetheless, these reality-based data are novel and valuable for future resource allocation in various settings. For example, preoperative preparation of TGM in the operative rooms for high-risk surgeries will reduce the time necessary for actual TGM application and contribute to patient safety. Further studies are warranted to determine the proper indication of TGM usage by proving the clinical benefit as well as cost-effectiveness when TGM is used in cases including the predictors identified in the present study.

## Conclusions

In conclusion, intraoperative TGM was used in 35.0% of spine surgeries of the present cohort, among which approximately one third involved unexpected usage. The predictors for intraoperative TGM usage included an ASA grade ≥ 2, rheumatoid arthritis, cervical spine, tumor, ligamentous ossification, posterior approach, durotomy, instrumentation, and osteotomy; furthermore, the female gender, ASA grade ≥ 2, cervical spine, tumor, posterior approach, durotomy, instrumentation, osteotomy, and microendoscopy-assisted surgery turned out to be predictors of unplanned TGM usage. In these cases as one expects increased or unexpected intraoperative bleeding that is difficult to control, preferential use of active hemostatic agents such as TGM may provide improved patient outcomes and decrease healthcare costs. While routine usage of TGM in these cases will require further justification, these novel findings are valuable for implementing preoperative precautions and optimizing resource allocation.

## Data Availability

The datasets used and/or analysed during the current study are available from the corresponding author on reasonable request.

## Abbreviations

TGM: Thrombin-gelatin matrix
CT: computed tomography
MRI: magnetic resonance imaging
ASA: American Society of Anesthesiologists
RA: Rheumatoid arthritis
BMI: Body mass index
